# Detection of Excess Presence of ^**99m**^Tc-MDP Near Injection Site—A Case Report

**DOI:** 10.3389/fmed.2021.728542

**Published:** 2021-09-17

**Authors:** James R. Crowley, Iryna Barvi, Debbie Greulich, Jackson W. Kiser

**Affiliations:** ^1^Department of Molecular Imaging, Carilion Clinic, Roanoke, VA, United States; ^2^Lucerno Dynamics LLC, Cary, NC, United States

**Keywords:** extravasation, infiltration, ^99m^Tc-MDP, injection quality, bone scans

## Abstract

Nuclear medicine extravasations and prolonged venous stasis may cause poor quality and quantification errors that can affect image interpretation and patient management. Radiopharmaceutical remaining near the administration site means that some portion of the radioactivity is not circulating as required for the prescribed uptake period. This case describes how detection of excess presence of ^99m^Tc-MDP near the injection site enabled the technologist to apply mitigation tactics early in the uptake process. It also suggests that detecting an extravasation or stasis early in the injection process can be important for image interpretation and minimizing radiation dose to tissue.

## Introduction

Bone scanning is a widely accepted diagnostic tool in nuclear medicine for many disease processes including metastatic screening. Bone scanning is typically a qualitative exercise, but as healthcare is rapidly moving toward precision medicine, quantification of molecular imaging techniques, including bone scans, is beginning to play a larger role. Quantification allows for more accurate and objective scan interpretation (1), and is becoming more important for bone scans when using new-generation treatments (2).

Extravasations, the leakage of the radiopharmaceutical into the tissue surrounding the administration site, frequently occur in bone scans. In nine centers, during two studies, 450 bone scan images were retrospectively reviewed for extravasations. The average extravasation rate was found to be 17.5% (3). Other nuclear medicine procedures are also frequently extravasated. A literature review revealed seven studies in 3 centers (2,989 patients) with an average extravasation rate of 15%^1^ (4–9) and that significant extravasations can affect the qualitative and quantitative assessment of nuclear medicine studies (1, 10–14). The experience at our center confirms the negative effects of significant extravasations. We previously published how an extravasation during an FDG PET administration resulted in understating the SUV_max_ of a solitary lung tumor by 80% (15). In the same case, we saw the effects of an extravasation to the quality of the image when we reimaged the patient 3 day later and discovered metastatic disease previously not evident in the image with extravasated injection. Using the extravasated image would have resulted in an improper treatment plan (15). In another publication, we highlighted the effects of significant extravasations on the quality and quantification of additional FDG PET studies and the quality of an MDP study by reimaging these patients several days after their extravasated studies. These cases also had patient care implications (16).

Prolonged venous stasis, a condition of slow blood flow in the vein (17), is another common problem in nuclear medicine. Accurate quantification requires a bolus injection of the entire intended dose. In the case of a prolonged venous stasis, the dose is not being delivered as a bolus; this delays the actual delivery of the radiopharmaceutical. Additionally, there is no information of how much of the dose may have not been delivered. Those two factors can confound the quantification of the scan.

Since extravasations and stasis can cause quantification errors, they should be considered when interpreting patient images. However, in many nuclear medicine studies the administration site is outside the imaging field of view (FOV) (4) and/or is not imaged for several hours after the injection, so physicians may not know that an extravasation or stasis occurred.

Significant extravasations can also potentially lead to high absorbed radiation doses to underlying tissue and skin (18). While bone scans and other routine diagnostic studies are often viewed as very low risk to patients, recent research indicates that significant extravasations of routinely used diagnostic radiopharmaceuticals like ^99m^Tc-MDP can result in high doses to the tissue and adverse tissue reactions (16, 18).

Detecting the excess presence of radiotracer immediately after administration can be important. Technologists, alerted that the injection quality may be compromised, can ensure the injection site is included in the field of view at time of imaging and can apply mitigation techniques early in the process. Early mitigation efforts for an extravasation or prolonged venous stasis can improve the image, minimize the absorbed dose, and decrease the latent effects of ionizing radiation to healthy tissue.

## Case Presentation

A 63-year-old female patient had a history of breast cancer and MRI showed an indeterminate lesion at L1. A SPECT/CT bone scan was ordered to rule out bone metastases.

**Figure 1:**
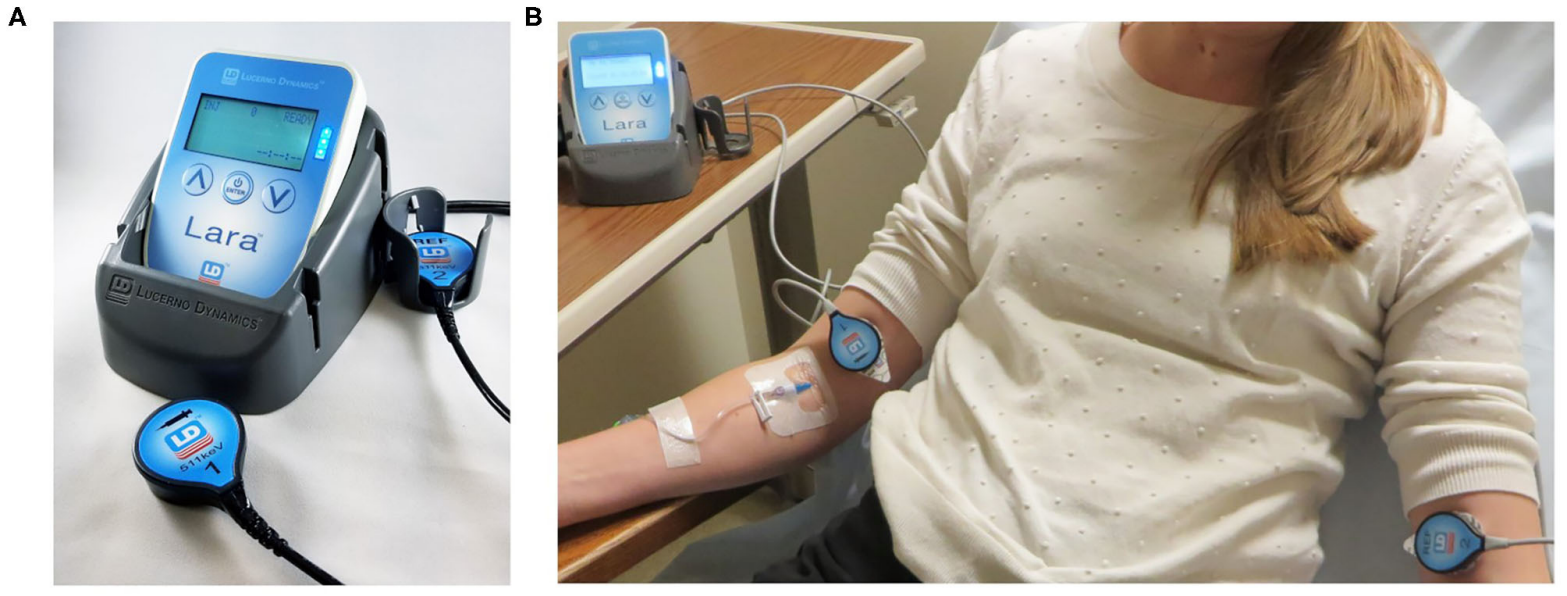
Lara device consists of 2 scintillation sensors, a reader, and a docking station **(A)**. Sensors placed on injection arm and reference arm **(B)**.

In our center, we monitor radiopharmaceutical injection quality using the Lara® System (Lucerno Dynamics, Cary, NC), consisting of two external scintillating detectors, a reader to collect and store data, a docking station, software to transfer data, and a web application to display and analyze data (Figure 1). The scintillating detectors are placed proximal to the injection site and mirrored on the other arm. The Lara® System provides a real time display of counts from each sensor and a time-activity curve (TAC) with a classifier score (19) once the administration is complete. Use of the device adds 20 additional seconds to the patient's imaging experience and <1 min/administration to the technologist's workload. A Certified Nuclear Medicine Technologist gained access to the right antecubital fossa using an IV cannula. The patient was manually injected with 27.2 mCi of ^99m^Tc-MDP and the IV was flushed with 10 mL of saline. Per protocol, the IV was then removed and the injection site then covered by a 2 × 2 gauze pad, which the patient held in place with firm finger pressure. Following a brief delay to ensure the site would not bleed, the gauze was wrapped with Coban to hold the gauze in place. The injection and IV removal were both unremarkable and presented no complications.

**Figure 2:**
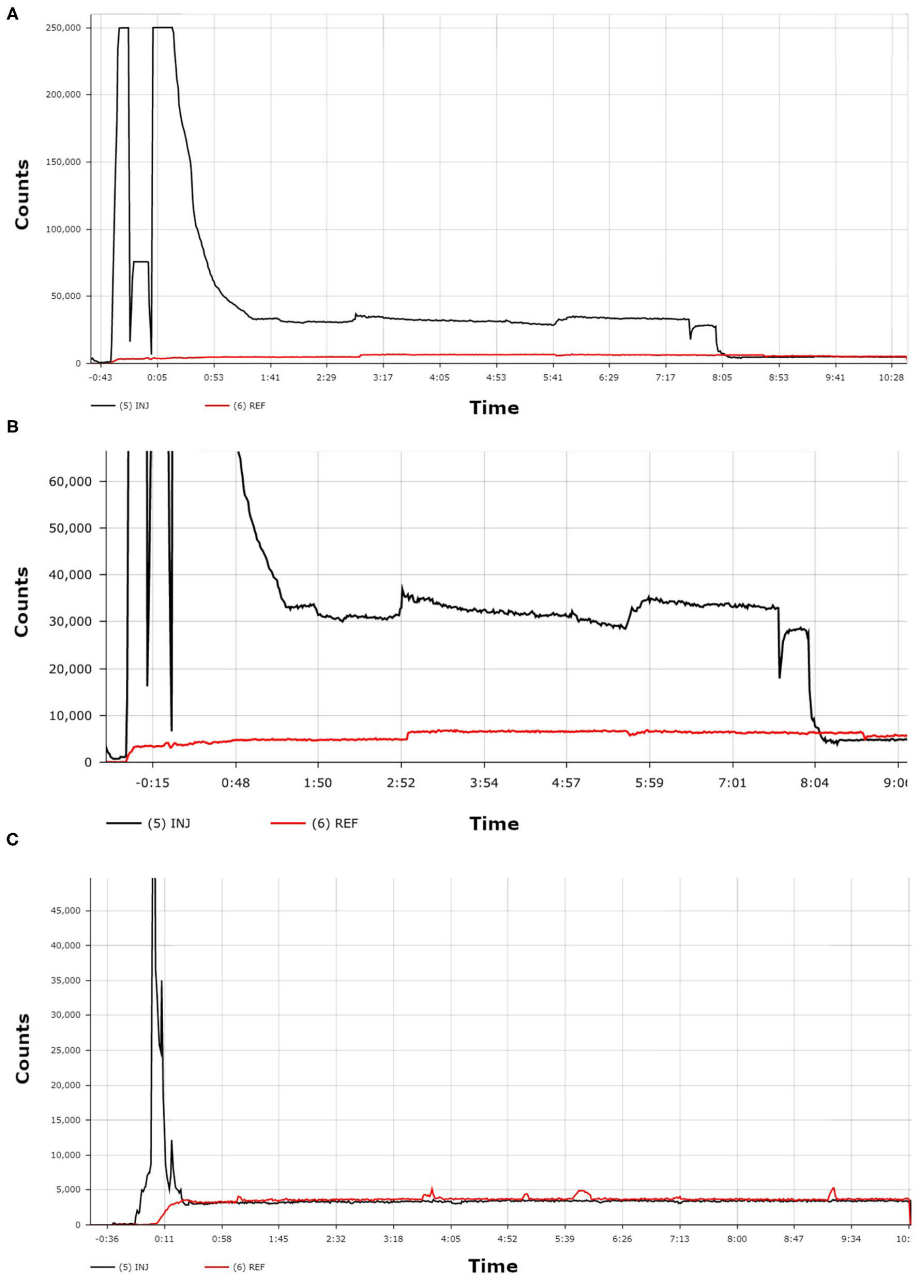
The first TAC **(A)** shows the patient's administration with ^99m^Tc-MDP. A zoomed-in view of this same TAC **(B)** more clearly shows the dramatic difference between the counts from the injection arm sensor curve (black line) and the counts from the reference arm sensor curve (red line). The third TAC **(C)** was acquired from a different patient and shows an ideal administration where the injection arm sensor curve (black line) and the reference arm sensor curve (red line) reached equilibrium within seconds.

While observing the patient, the technologist checked the Lara reader display. Counts from the sensor on the injection arm, as compared to counts from the reference sensor, indicated a significant presence of excess ^99m^Tc-MDP near the injection site (Figure 2). The technologist asked the patient to move her injection arm and to leave it raised above her head for several minutes. The technologist observed the real-time counts on the Lara display begin to decrease from the injection sensor, indicating the clearance of the MDP from the injection site. Over the next 8 min the counts from the injection sensor equilibrated with the counts from the reference sensor. The uptake period resumed with no further issue and the patient was imaged according to our standard protocol.

Bone SPECT images were acquired with a Siemens Intevo Bold (Siemens Medical Solutions USA, Inc.) after standard injected activity of 27mCi ^99m^Tc-MDP using a LEHR collimator and matrix size of 256 x 256 with a zoom of 1. SPECT data were acquired using 30 views in a non-circular (body contour) orbit with an acquisition time of ~20 s per view. For CT Acquisition, data were acquired using 130 kVp with CareDose 4D and pitch of 1.5 and detector settings of 16 × 1.2 mm with 3 mm slice thickness.

## Discussion

Without monitoring the quality of the administration, extravasation and stasis are difficult to detect during and after radiopharmaceutical administration for several reasons. First, nuclear medicine scans usually use small injection volumes of non-vesicant radiopharmaceuticals that do not cause immediate, visible changes to the overlying skin near the injection site, nor immediate pain to the patient. Second, during clinician interpretation of the images, the injection sites are often outside of the imaging FOV. Even if the injection site is in the imaging FOV, prolonged venous stasis is especially confounding since at the time of imaging there may be no evidence at the injection site that there had been a venous issue during the administration of the radiopharmaceutical (20).

In the case when an extravasation is visible, many centers use lead shielding to prevent artifacts. While this beneficial practice prevents artifacts from confounding image quality, it does not address quality issues associated with a reduced number of counts in the imaging area of interest. This unknown impact to the quality of the image is more pernicious because there is a lack of knowledge regarding how much activity remains near injection site.

Application of external scintillation detectors to monitor activity at the injection site provides dynamic insight into the quality of radiopharmaceutical administration without having to image the injection site and can assist the interpreting physician to determine if repeat imaging is needed. In a case of extravasation, monitoring with external scintillation detectors also provides patient-specific biological clearance information to perform more accurate dosimetry (18).

Prospective monitoring also enables early mitigation efforts. Mitigation steps can include one or more of the following: local application of warmth, elevation or movement of arm laterally, massaging, and flushing with saline (17, 21, 22). The purpose of these interventions is to either resolve a stasis or minimize the effects of an extravasation. Mitigation efforts increase blood flow and lymphatic clearance and disperse radioactivity. While some references suggest applying ice to a radiopharmaceutical extravasation (23), this will constrict blood flow and possibly retain radioactivity locally. Additionally, certain vascular access techniques prevent some mitigation tactics; it is impossible to flush saline if straight sticks have been used for vascular access.

This case report has limitations. At our center bone scan uptake periods often last for 3 h and we do not require that patients remain still. This prolonged stasis may have resolved itself sometime during the patient's 3-h uptake period without monitoring and then applying mitigation steps.

## Results

**Figure 3:**
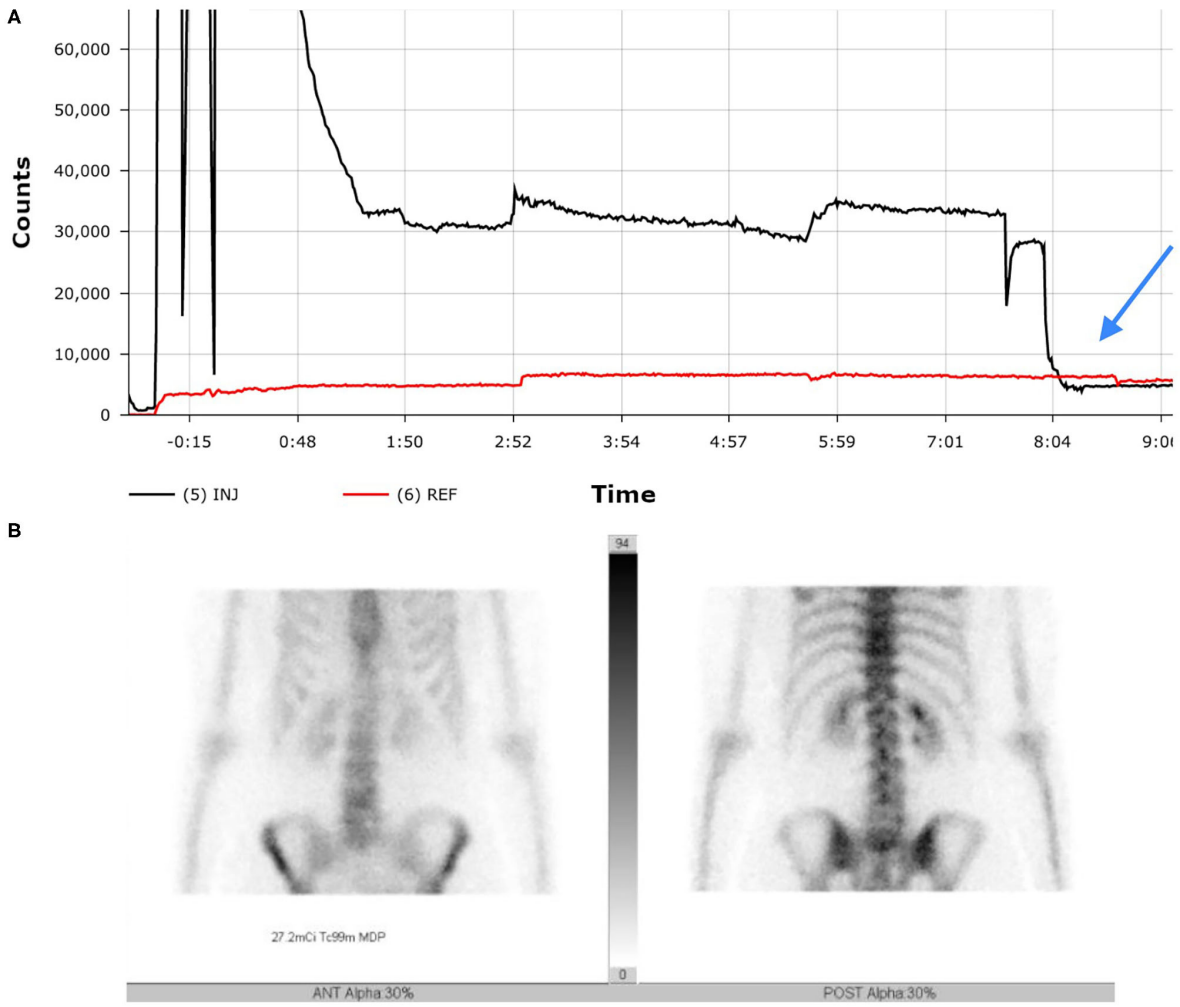
While some excessive level of radioactivity is indicated in the first few minutes of uptake on the TAC **(A)**, the mitigation efforts at time 8 minutes cause rapid equilibrium (see blue arrow). This equilibrium is reflected in image **(B)** no remaining radioactivity evident at injection site.

During the standard imaging protocol for the study, additional bilateral antecubital static images in both anterior and posterior views were also acquired (Figure 3). The images revealed no evidence of remaining radioactivity in the right antecubital fossa, leading us to the conclusion that by having the patient raise the right arm above their head, the radiopharmaceutical was able to completely infuse into circulation resulting in an uncompromised study. The SPECT-CT bone scan showed that the patient had benign hemangioma with no uptake of radiotracer or any other signs of metastatic skeletal disease. The patient has had no further imaging evaluation since then regarding her cancer except for routine mammography.

## Concluding Remarks

This case describes how an early detection of excess presence of ^99m^Tc-MDP near injection site enables technologist to apply mitigation tactics early in the process. It also suggests that detecting an extravasation or stasis early in the injection process can be important for image interpretation and minimizing radiation dose to tissue.

## Data Availability Statement

The original contributions presented in the study are included in the article/supplementary material, further inquiries can be directed to the corresponding author.

## Ethics Statement

Ethical review and approval was not required for the study on human participants in accordance with the local legislation and institutional requirements. The patients/participants provided their written informed consent to participate in this study.

## Author Contributions

JC contributed by obtaining patient data and preparation of the manuscript. IB and DG contributed to preparation of the manuscript. JK contributed by interpreting patient data and preparation of the manuscript. All authors contributed to review, editing, read, and approved the final manuscript.

## Conflict of Interest

IB and DG are employed by the company Lucerno Dynamics, the manufacturer of the Lara System described in this manuscript. The remaining authors declare that the research was conducted in the absence of any commercial or financial relationships that could be construed as a potential conflict of interest.

## Publisher's Note

All claims expressed in this article are solely those of the authors and do not necessarily represent those of their affiliated organizations, or those of the publisher, the editors and the reviewers. Any product that may be evaluated in this article, or claim that may be made by its manufacturer, is not guaranteed or endorsed by the publisher.

## Footnotes

^1^Meyer A, Higgins Z, Packard A, Johnson D. Importance of Injection Site Image in DaTscans. Mayo Clinic, Department of Radiology, Rochester, Minnesota.

## Abbreviations

^99m^Tc-MDP: Technetium 99m-methyl diphosphonate
mCi: Millicurie
SPECT/CT: Single Photon Emission Computed Tomography/Computed Tomography
TAC: Time-activity curve
FOV: Field of view
IV: Intravenous
mL: Milliliter.
